# Optimization of Titanium Dental Mesh Surfaces for Biological Sealing and Prevention of Bacterial Colonization

**DOI:** 10.3390/ma15072651

**Published:** 2022-04-04

**Authors:** Nuno Cruz, João Paulo Tondela, Maria Inês Martins, Eugenio Velasco-Ortega, Javier Gil

**Affiliations:** 1 School of Dentistry, Universitat Internacional de Catalunya (UIC), C. Josep Trueta s/n, 08195 Sant Cugat del Vallès, Spain; nuno.cruz@orismed.pt; 2 Bioengineering Institute of Technology, International University of Catalonia, Josep Trueta s/n, 08195 Barcelona, Spain; 3 CIROS from the Faculty of Medicine, University of Coimbra (FMUC), Av. Bissaya Barreto, 3000-075 Coimbra, Portugal; 4 Faculty of Engineering, University of Porto (FEUP), 4200-465 Porto, Portugal; up201305982@fe.up.pt; 5 Master in Implant Dentistry, Faculty of Dentistry, University of Seville, c/Avicena s/n, 41009 Seville, Spain; evelasco@us.es

**Keywords:** dental meshes, titanium, sealing, bacteria, roughness, wettability

## Abstract

Titanium dental meshes have a wide application in order to ensure the retention of calcium phosphate-based biomaterials to regenerate bone tissue. These meshes are temporary and must grow a soft tissue to prevent bacterial colonization and provide stability. In this work, we aimed to optimize the roughness of the meshes to obtain a good biological seal while maintaining a behavior that did not favor bacterial colonization. To this end, six types of surfaces were studied: machined as a control, polished, sandblasted with three different alumina sizes and sintered. The roughness, contact angles and biological behavior of the samples using fibroblast cultures at 7, 24 and 72 h were determined as well as cytotoxicity studies. Cultures of two very common bacterial strains in the oral cavity were also carried out: *Streptococcus sanguinis* and *Lactobacillus salivarius*. The results showed that the samples treated with alumina particles by sandblasting at 200 micrometers were the ones that performed best with fibroblasts and also with the number of bacterial colonies in both strains. According to the results, we see in this treatment a candidate for the surface treatment of dental meshes with an excellent performance.

## 1. Introduction

Currently, modern oral rehabilitation focuses on minimally invasive approaches in order to obtain the desired results with regard to the patient’s best interests and least discomfort as possible.

However, when it comes to oral surgery involving soft and hard tissue reconstruction, the idea of a minimally invasive approach relies on the complexity of the diagnosis and correspondent treatment plan(s). Moreover, when complex bone losses, combining horizontal and vertical defects, or atrophic maxillary/mandibular need volume improvements in order to rehabilitate function and aesthetics with fixed teeth, we can only expect higher challenges that end in a more demanding treatment for both patient and clinician.

The success of dental implant treatments depends on the bone quality as well as the alveolar volume for the proper placement of dental implants. Implant design, biomaterials, good primary fixation with cortical bone and good soft tissue healing also play important roles [1].

Permanent prostheses fixed by dental implants for the rehabilitation of the oral cavity do not mean a simple fitting following the shape of the bone tissue. Currently, surgeons must make treatment plans for each patient and must analyze bone defects, determining suitable spaces for placement via computed tomography (CT)/cone beam computed tomography (CBCT) [2].

Several studies have been conducted to regenerate alveolar bone tissue after tooth loss. These surgical regeneration procedures will be the key to the success of the treatment [2,3]. These studies report that almost half of rehabilitations with dental implants need some type of bone regeneration procedure. These procedures can be performed before or during the implant placement [3].

Different methodologies and clinical procedures have been developed to restore bone defects in the alveolus. One of the most prominent is guided bone regeneration (GBR) as well as bone grafting, bone extrusion and new bone generation by distraction.

Due to the simplicity of the technique, its ability to create new bone tissue in different directions and its stability, GBR is currently the most widely used technique for the repair of alveolar bone defects [4]. The resorbable barrier membrane (RBM) technique consists of preventing epithelial cells and connective tissue cells from proliferating in the area of the bone defect by using the barrier membrane. The different migration rates of the different cells allow osteoblastic cells to preferentially enter the bone defect area to induce and regenerate new bone tissue [5].

There are two different types of barrier membranes used, in most cases, for barrier membrane (BR): resorbable or non-resorbable. Both have the same mission, which is to act as a mechanical barrier to calcium phosphate-based materials. These BRs differ in chemical composition and macro- and microdesign, but they always have the same retentive function [6]. To fulfil this main objective, the meshes must have good biocompatibility and mechanical strength in order to have a good retentive capacity [7].

In the presence of significant bone tissue defects, both vertical and horizontal, titanium or Ti6Al4V alloy meshes are ideal, as demonstrated by several clinical studies, given their good mechanical properties and osteogenic capacity [8,9,10].

Recently, new designs of titanium mesh membranes and titanium alloys (Ti6Al4V) have been studied with the aim of facilitating the formation of new bone tissue, stabilizing bone grafts beneath the membrane while minimizing the possibility of fibrous tissue growth and/or preventing collapse [11,12].

The optimal membrane should facilitate cell activity (i.e., adhesion, proliferation, migration and differentiation cellular) on the membrane surface in order to isolate the defect from the presence of bacteria, in addition to the main function of the membrane. This biological sealing produced by the cells of the connective tissue will stabilize the blood clot causing the integration of the soft tissue into the membrane. However, fibroblasts must be prevented from penetrating the membrane, since it could be biologically harmful [13].

In addition to its good biocompatibility and mechanical properties, titanium and Ti6Al4V also has excellent corrosion behavior due to the formation a passive and inert oxide film [14,15]. In addition, the reconstruction of an alveolar ridge, a thin 1–2 mm thick soft tissue layer, produced by the metallic mesh can be observed on the regenerated bone tissue on the surface, called the “pseudoperiosteum”. The mission of the pseudoperiosteum is bone graft protection, or prevention of graft bacteria colonization [16].

However, exposure rates and consequent healing complications due to the use of titanium meshes in bone regeneration remains a major concern. The incidence of mesh exposure is mostly 20 to 30%, and the highest reported exposure rate is 66% [17,18,19,20].

As is known, primary wound closure and soft tissue stability during the remaining healing period play crucial roles in avoiding early and late exposure of the titanium mesh. As mentioned, the differences in the superficial properties and the porosity characteristics (i.e., number, sizes and distribution) may produce different behaviors in cell adhesion, migration, proliferation and differentiation. Furthermore, it is suggested that cell adhesion onto surfaces is produced by protein interactions in the body environment and that the properties of this layer depend on characteristics such as surface electrical charge, chemical elements and the internal energy of the titanium [21,22].

The results of Rakhmatia and collaborators’ work evaluated the difference in fibroblast adhesion and morphology in relation to the exposition of different designs and structures of GBR barrier membranes [23]. Several factors, such as membrane material, topography, design, adhesion behavior, protein-binding ability, debris released during degradation, wettability, internal energy, texture and duration of barrier function, may influence GBR outcomes, which has not yet been completely understood [24].

The aim of this contribution is to determine the best conditions of roughness of the surface of the meshes so that they perform the barrier function, and for this there is a good adhesion, proliferation and differentiation of fibroblasts in order to obtain a good biological seal that prevents bacterial colonization. It is also important that the topography does not favor the activity of osteoblastic cells to prevent osseointegration. Once these meshes have performed their function of bone regeneration, they must be removed and, therefore, it is not good for them to remain anchored in the bone tissue. For these reasons, the best conditions for the surface topography of the meshes should be studied, which we aimed to clarify in this work.

## 2. Materials and Methods

### 2.1. Materials

**Figure 1:**
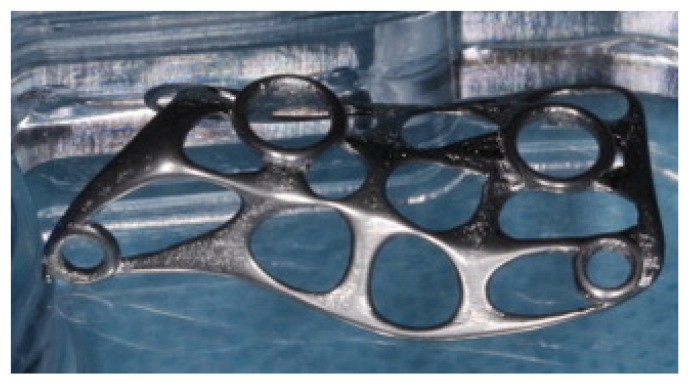
Ti6Al4V mesh used in this study.

One hundred and twenty grade 5 titanium alloy (Ti6Al4V) meshes (BoneEasy, Arada, Portugal) were used. Figure 1 shows the mesh design used.

Cylindrical shape samples (5 mm diameter, 2 mm width) were cut, and six different surfaces were evaluated:

- (Mech): As-received lathe-cut titanium samples (i.e., control samples). The Mech samples used in the study corresponded to the same material, roughness and mesh conditions as shown in Figure 1. The samples were extracted from the same material with the same mesh conditions;
- (Smooth): samples were treated with 220 to 4000 grit SiC paper in water medium, deburred and after polished with SiO_2_ suspension.

Sand-blasted: the surfaces were sand-blasted at a pressure of 2.5 MPa with:

- (Al2): Al_2_O_3_ small size particles (212–300 μm);
- (Al6): Al_2_O_3_ medium size particles (425–600 μm);
- (Al9): Al_2_O_3_ large size particles (1000–1400 μm);
- (Sinter): Ti6Al4V spheres sintered from 10 to 50 μm in diameter.

After treatment, all samples were cleaned with deionized water, ethanol and acetone; dried at 25 °C; sterilized by autoclave at 120 °C for half an hour.

### 2.2. Characterization of the Surfaces

Roughness parameters were obtained by means of a white light interferometer microscope (Wyko NT1100, Veeco Instruments Inc., Tucson, AZ, USA) and proprietary software (Vison32, Veeco Instruments Inc., Tucson, AZ, USA). The measurements were realized in 10 samples to determine the average roughness (Ra), which represents the mean height of the peaks indicated by the arithmetic average of the absolute values of all points of the profile, and the real surface area (Ar), larger than the nominal area (70.7 mm^2^) due to the surface roughness.

Hydrophilic and hydrophobic characters were measured using a contact angle video-based system (Contact Angle System OCA15plus, Dataphysics, Filderstadt, Germany) and analyzed with proprietary software (SCA20, Dataphysics, Filderstadt, Germany). The analysis was performed under conditions of 100% relative humidity and controlled temperature.

The topography of the samples was observed by scanning the electron microscopy (SEM) using the Phenom XL Desktop SEM microscope (PhenomWorld, Eindhoven, The Netherlands) using a voltage of 20 keV to accelerate the electrons and to achieve a good resolution (7 nm). This microscope can perform EDX microanalysis in order to conduct atomic chemical analysis with a sensitivity of approximately 0.1%.

### 2.3. Cell Culture and Cell Seeding

Primary human foreskin fibroblast cells (Millipore, Billerica, MA, USA) were cultured in Dulbecco’s minimal essential medium (DMEM; Invitrogen, Carlsbad, CA, USA) with the addition of 10% fetal bovine serum (FBS), L-glutamine (2 mM) and penicillin/streptomycin (50 U/mL and 50 g/mL, respectively) at 37 °C in a humidified incubator at 5% CO_2_. The culture medium was changed every 48 h. Subconfluent fibroblasts were trypsinized, centrifuged and seeded at 6 × 10^3^ cells/disc with DMEM without serum and phenol red in the different Ti6Al4V samples and placed in a 48-well microplate. An agarose film was introduced (in order to inhibit fibroblast adhesion) in order to have a negative control and determine the adhesion behavior. Tissue culture polystyrene (TCPS) and polished Ti6Al4V (Smooth) were used as reference substrates. Fibroblast analyses were carried out at 4, 24 and 72 h.

### 2.4. Cell Morphology

Field emission scanning electron microscopy (FESEM) (JSM-7001F JEOL Ltd., Tokyo, Japan) was used to characterize the cellular morphologies. For this objective, the cultured discs were cleaned by means of 0.1 M phosphate buffer (PB) and fixed with 2.5% glutaraldehyde solution in PB for 4 h at 4 °C. The samples were immersed for 2 h at room temperature in a 1% solution of osmium tetroxide in order to improve the observation. Fixed samples were then dehydrated in 50, 70, 90, 96 and 100% ethanol series three times followed by a hexamethyldisilazane (HDMS) drying procedure.

### 2.5. Cell Proliferation—WST-1

HFF fibroblasts were cultured on the different surfaces studied, analyzing adhesion and proliferation using WST-1 (Roche Applied Science, Penzberg, Germany). This colorimetric determination quantifies cell activity by formazan staining. The mechanism is that mitochondrial dehydrogenases in living cells cause the separation of tetrazole salts, and the color of the soluble formazan is measured spectrophotometrically. The absorbance increases and can be correlated with increasing cell number. Cell viability was determined at the different specified culture times by incubating for 2 h with WST-1 1:10 in DMEM without serum and phenol red. The optical density (OD) at 440 nm of the cell supernatant was measured with the ELx800 universal microplate reader (Bio-Tek Instruments, Inc., Winooski, VT, USA). Three different samples were studied for each surface type, and two different experiments were performed in parallel. The optical density (OD) at 440 nm of the cell supernatant was determined with the ELx800 universal microplate reader (Bio-Tek Instruments, Inc., Winooski, VT, USA). Three samples were studied for each surface type, and two tests were performed. A curve was obtained using different numbers of cells from 3 × 10^3^ to 50 × 10^3^.

### 2.6. Cell Viability—LDH

Lactate dehydrogenase (LDH) enzyme release at culture times was the methodology used for quantification of non-viable cells. The supernatant liquid was extracted from the cell-free culture. This broth was centrifuged at 250× *g* for 5 min and subsequently detected by the Cytotoxicity Kit LDH (Roche Applied Science, Penzberg, Germany). The decrease in tetrazolium compounds in formazan staining by LDH activity was determined spectrophotometrically using 490 nm. TCPS was used as a minimum control and lysed cells (maximum LDH activity) as a maximum control. Two experiments were realized in order to evaluate the cytotoxicity of the three samples in each series.

### 2.7. Microbiological Behavior

The bacteria strains *Streptococcus sanguinis* (CECT 480) and *Lactobacillus salivarius* (CECT 4063) (Colección Española de Cultivos Tipo, Valencia, Spain) were tested in this research. Strains were cultured in Todd–Hewitt broth at 37 °C in a 5% CO_2_-enriched atmosphere. The microbial adhesion to solvents (MATS) assay [25] was followed to determine bacterial adhesion in physiological medium; the MATS test is based on the electronic exchange of bacteria (donor/acceptor) [26,27,28].

Bacteria were collected when proliferation was in the exponential growth function. Bacteria were collected after centrifugation at 4500× *g* for 15 min at a temperature of 4 °C. Once obtained, the bacteria were washed with phosphate buffer solution (PBS) at 0.15 M. The bacteria were then suspended in PBS and their optical density was determined at a wavelength of 550 nm (A_0_). The MATS test was performed in hexane, chloroform and diethyl ether. Three microliters of bacteria dissolution were extracted into 9 tubes, and 400 μL of solvent (3 samples for each solvent) were added. The different suspensions were incubated at 20 °C for 10 min and mixed in a vortex shaker (Scientific Industries, Bohemia, NY, USA) for 1 min. Phase separation was performed after 15 min by measuring the optical density of the aqueous phase at the same wavelength (A_1_). The resulting bacterial adhesion was determined according to the formula: (1 − A_1_/A_0_) × 100.

Ti6Al4V samples of 5 mm in diameter and 2 mm in thickness were tested. These were cleaned in 70% ethyl alcohol, acetone and distilled water, dried at room temperature and autoclaved. These discs were seeded with two bacterial strains that are frequently present in the oral cavity: *Streptococcus sanguinis* (CECT 480) and *Lactobacillus salivarius* (CECT 4063). The bacteria were incubated on the discs for 2 h at 37 °C and 5% CO_2_. Subsequently, they were washed with PBS and detached in Ringers’ solution. Bacterial seedings from the suspension (MRS for *Lactobacillus salivarius* and Todd–Hewitt for *Streptococcus sanguinis*) were incubated at 37 °C for 2 days. Subsequently, the number of colonies was analyzed. The variation in acidity during bacterial growth was also determined.

The discs were cleaned with phosphate buffer (PB, pH 7.2–7.4) for 5 min and then fixed with a 2.5% solution of glutaraldehyde in 0.1 M PB for 30 min at 4 °C. This washing process was repeated twice. After washing for 5 min with PB thrice, the discs were stored at 4 °C and prepared for further treatment according to the MATS.

The samples were dehydrated by 10 min of exposure to a graded sequence of aqueous ethanol (30–100%) and, finally, dried overnight at 25 °C. Then, the discs were treated by sputtering in order to coat with a carbon (Emitech k950x, Kent, UK) and could be observed by SEM. 

### 2.8. Statistical Analysis

Data are expressed as the mean ± standard deviation. Statistical analysis was performed using MINITAB^®^ (version 18, Minitab Inc., State College, PA, USA). We used nonparametric tests, because although the normal distribution of each data population was confirmed by the Anderson–Darling normality test, homoscedasticity was ruled out (Barlett and Levene’s test for homogeneity of variances). Therefore, we used the Kruskal–Wallis test for multiple comparisons and the U Mann–Whitney test for individual (one-to-one) comparisons. Statistical significance was set at *p* < 0.01.

## 3. Results and Discussion

### 3.1. Surface Characterization

**Figure 2:**
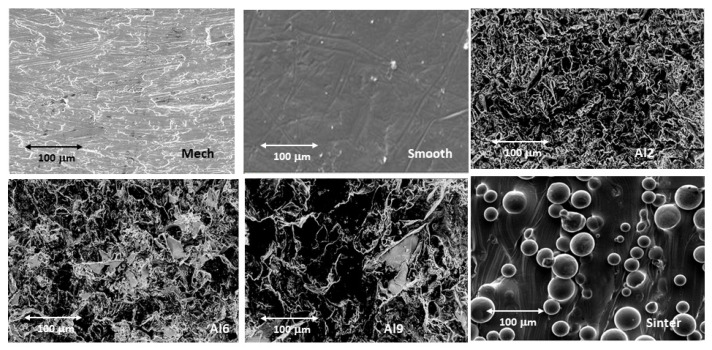
Surfaces observed by scanning electron microscopy with the same magnification for each treatment studied.

**Figure 3:**
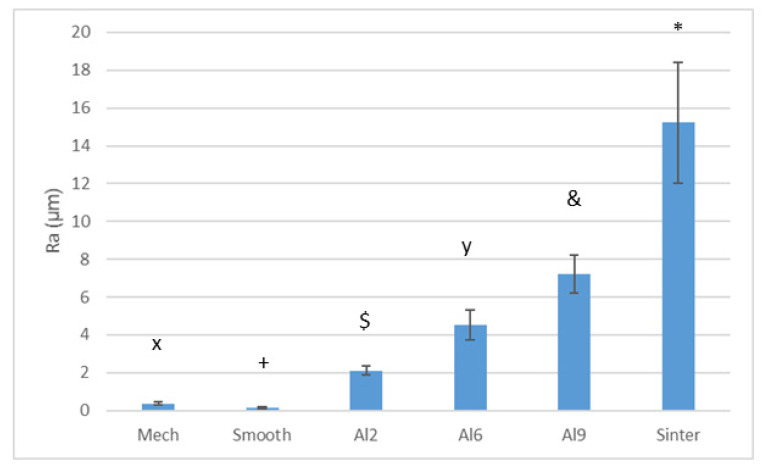
Roughness (Ra) obtained by each treatment. Each symbol means that the results were different with statistical significance to the other symbols. All the results present difference with statistical significance at *p* < 0.01. For the Ra values, the different surface treatments showed roughness values that were all statistically different from each other.

Figure 2 shows the studied surfaces observed by electron microscopy. From this figure, it can be seen that the values with the lowest roughness were the polished samples, and an increasing roughness can be seen as the size of the abrasive alumina particles used in the sandblasting process increased. Small abrasive particles (i.e., Al2) produced Ra ≈ 2.02 μm, whereas other particle sizes (i.e., Al6 and Al9) obtained Ra ≈ 4.21 and 7.10 μm. Likewise, the surface of the sintered samples could be observed on the Ti6Al4V surface, showing that the welding processes of the spheres were not very severe, as they maintained the morphology of the spheres at approximately 80% of the initial volume of each sphere. In this case, the roughness was higher than 14 μm. The roughness values obtained are shown in Figure 3, where all the surfaces presented statistically significant differences between them with a *p* < 0.01.

**Figure 4:**
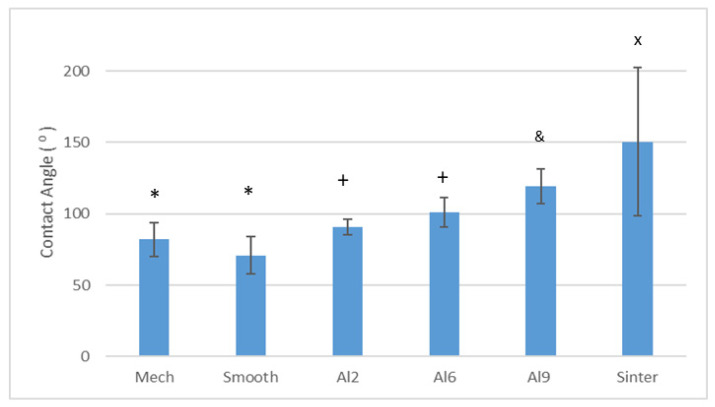
Contact angles obtained by each treatment. Each symbol means that the results were different with statistical significance to the other symbols at *p* < 0.01. The contact angles of the Sinter and Al9 samples showed statistically significant differences between all other surfaces. The Mech and Smooth samples did not show significant differences between them, but they did show significant differences between all the other samples. The same was true for the Al2 and Al6 samples, which did not differ statistically from each other but did differ from the rest of the samples.

The contact angles results are shown in Figure 4. The contact angle values presented good correlation with roughness: Smooth and Mech samples (Smooth Ra ≈ 150 nm and Mech Ra ≈ 360 nm) presented a contact angle ≤80°, while Al2 (Ra ≈ 2.02 µm) presented a result of ≈90°, and Al6 and Al9 presented results of ≥98°. The very high values of the contact angles of the sintered mesh, reaching values of 150° (i.e., a very hydrophobic character), are noteworthy. This important difference could be due to the fact that the sintering treatment requires reaching very high temperatures, producing microstructural changes in the Ti6Al4V which, in addition to producing an important grain growth, causes a change in the structure from mill annealed to Widmasntatten structures [29,30,31]. These structural changes could justify this important increase in the contact angle.

Table 1 shows the atomic compositions of the different samples studied. Ten measurements were performed for each. The slight presence of aluminum can be observed in the samples that were sand blasted with alumina. Moreover, in the titanium, some traces of iron can be observed, which is a common impurity in medical-grade titanium. It can be said that the samples did not have a clean surface with very little contamination.

### 3.2. Cell Proliferation and Cytotoxicity

**Figure 5:**
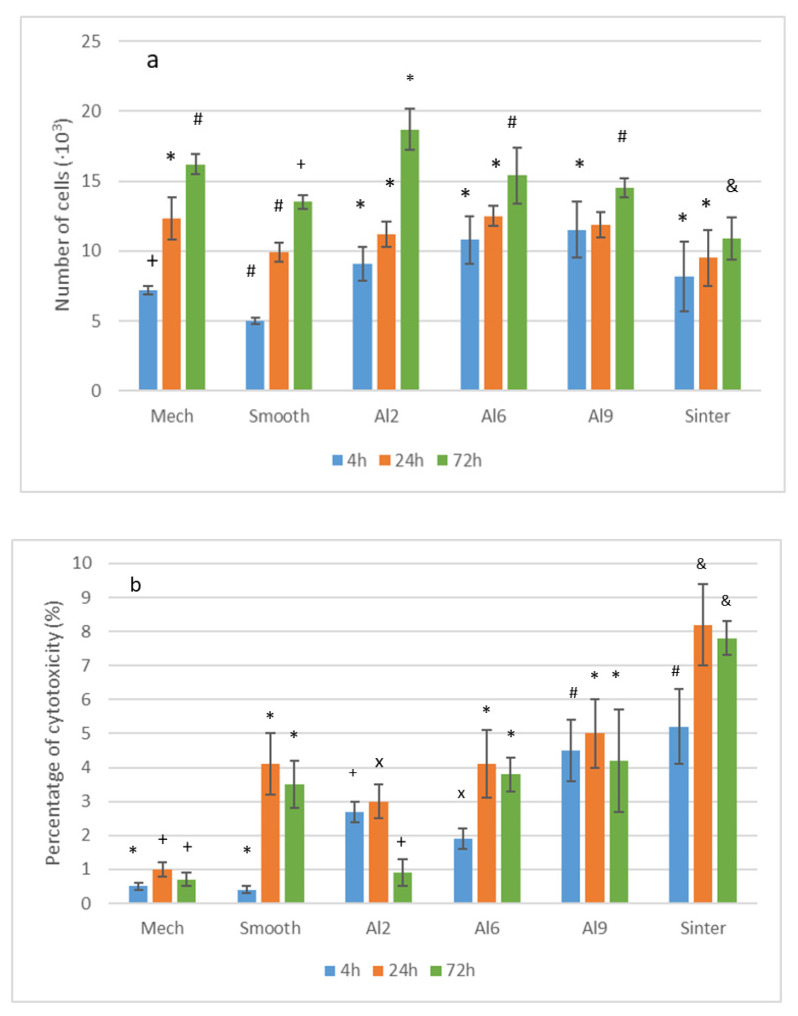
Analysis of cell viability onto the different micromachined and reference surfaces at 4, 24 and 72 h: (**a**) WST-1 cell proliferation tests; (**b**) released LDH activity demonstrated that cytotoxic effects were less than 10% of the positive control for all the tested surfaces in all the times analyzed. Each symbol means that the results for each time were different with statistical significance to the other symbols at *p* < 0.01. The statistical study was carried out for the six types of surfaces for the three times studied. The differences were established for each time.

Fibroblast proliferation was quantified measuring the conversion of tetrazolium salts into soluble formazan dye by metabolically active cells. HFFs were cultured onto different surfaces, and the absorbance at 440 nm after WST-1 addition was measured at 4 h and 1 and 3 days after cell seeding. A standard curve using serial dilutions of cell numbers was prepared to extrapolate absorbance sample values. The number of living cells after the different times for each surface can be seen in Figure 5a. As can be observed, after 4 h of culture, there were no statistically significant differences in cell viability between any of the tested surfaces. In addition, after 72 h, there were statistically more living cells on Al2 surfaces than the other surfaces showing a statistically difference significance (*p* < 0.01).

The behavior of Al2, in which cell proliferation did not stand out over a short time period, was due the fact that on that surface the adhesion and proliferation processes proceeded very fast, and at those times the cells were already in the differentiation phase. Subsequently, an increase in the number of living cells was observed [32]. Therefore, we can say that the Al2 surface was the one that showed the best behavior towards biological sealing.

As is well known, in dental meshes we need soft tissue to cover the mesh to prevent bacterial colonization and also to prevent osteoblastic cells from adhering. If osteoblastic adhesion, proliferation and differentiation were to occur, the mesh could become osseointegrated, and it would be difficult to remove the plaque once the bone regeneration biomaterial had succeeded in increasing the bone volume. This is why the mesh needs the formation of soft tissue that seals the dental mesh from bacterial attack and that this tissue forms quickly to avoid the formation of bone tissue that would make mesh removal difficult. The dentist himself removes it once the bone has regenerated.

The first step in cell adhesion to a surface is the key role of cell viability. This process depends not only on the surface chemistry but also on the surface roughness. Despite the fact of its biocompatibility, it has been demonstrated that fibroblasts adhere better to sandblasted samples than to Mech and Smooth samples. Although, several studies have been realized on the generation of micro- and nano-roughness in order to induce cell orientation; nevertheless, a better adhesion to modified titanium was not demonstrated. Our results demonstrated that cells adhere better and proliferate earlier on Al2 surfaces compared with the other tested surfaces. Moreover, these results suggest that initial adhesion was more related to micro-roughness.

Cytotoxicity was assessed measuring the reduction in tetrazolium salts into formazan dye by LDH activity released by damaged cells. In Figure 5b, it can be observed that the cytotoxicity for the different surfaces was studied. Although there were only statistical differences between the roughened and Smooth surfaces at 4 h, the cytotoxicity was below 10% of the positive control (Mech) result for the different times and types of surface. In no case did the surfaces show cytotoxicity, the most compatible surfaces being those with the least roughness. It is worth noting that the significant difference in the sintered meshes, which reached values of almost 9%, probably due to the internal stresses caused by the welding, although this did not affect the good biological behavior with the fibroblast cells [33,34].

**Figure 6:**
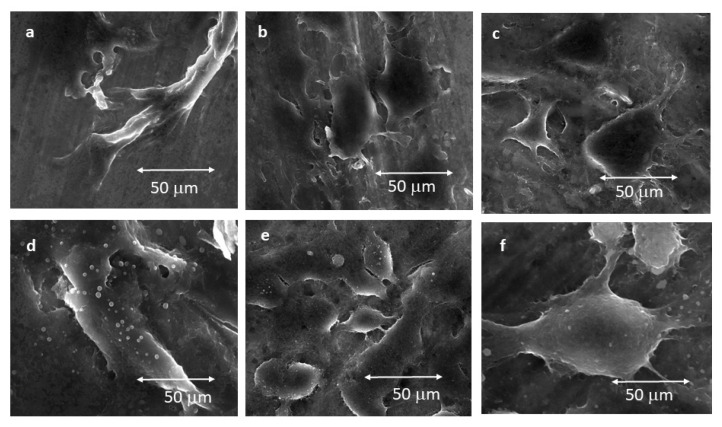
FESEM images of fibroblasts morphologies: (**a**) Mech samples at 4 h; (**b**) Al2 samples at 4 h; (**c**) Al9 samples at 4 h; (**d**) Mech samples at 72 h; (**e**) Al2 samples at 72 h; (**f**) Al9 samples at 72 h.

FESEM observations showed that fibroblasts were flattened, and their distribution did not show any preferred orientation when they were cultured on Mech and Smooth titanium after 4 h of culture (Figure 6a). On the other hand, for the micro-roughness surfaces, Al2, Al6 and Al9, after 4 h after cell seeding the fibroblasts presented an elongated shape and were placed in the valleys. Moreover, cells attached on the Al2 series accommodated entirely inside the valleys, presenting a semi-flattened morphology (Figure 6b), whereas for the Al6 and Al9 series, the cells grew up occupying part of the ridges (Figure 6c). At higher magnifications, it was observed that fibroblast cells adhered to the titanium by filopodia-type digitations. In Figure 6d–f, the morphologies of the fibroblasts after 72 h for Mech, Al2 and Al9 can be observed, respectively. It can be seen that the cells were rounded and had different filopodia between the fibroblasts forming the soft tissue.

Subsequently, the possibility of the modified surfaces activating the seeded fibroblasts was analyzed. In physiological and pathological situations, such as wound healing, fibroblasts were recruited at the injured site, and they were activated to a transient state called myofibroblast. In this state, they expressed α-SMA, a characteristic marker of smooth muscle cells that confers cytoskeleton contractility and synthesizes and remodels the extracellular matrix (ECM) until they resolve the wound [35]. After that, it is suspected that myofibroblasts disappear, mainly via apoptotic pathways induced by the mechanical load of the reconstructed ECM [36], and resident fibroblasts colonize the healed zone and proliferate. Otherwise, persistent myofibroblast proliferation and/or survival are considered an aberrant ECM repair that leads to a fibrotic disease or wound repair failure [37]. Meanwhile, after biomaterial implantation, fibroblasts must be activated to promote fibrointegration. Fibroblasts that colonize the biomaterial, initially adhered by proteins adsorbed on it, activate to a myofibroblastic phenotype and start to remodel and secrete their own ECM until this reconstructed ECM induces their apoptosis. Nevertheless, if this process fails, it could fall into implant lost.

### 3.3. Microbiological Behavior

**Figure 7:**
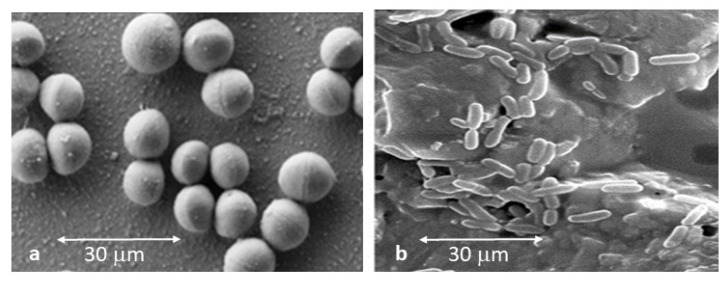
Bacteria strains tested in this research: (**a**) *Streptococcus sanguinis*; (**b**) *Lactobacillus salivarius*.

Samples were observed by scanning electron microscopy to determine if bacteria adhered onto the different Ti6Al4V surfaces. Figure 7a shows the usual morphology of “necklace of pearls” of *Streptococcus sanguinis*; this shape demonstrates the adhesion onto the Ti6Al4V surface. The observed size of the bacteria ranged from 0.7 to 2.2 micrometers in diameter. It is common for two *Streptococcus sanguinis* to join at the hemispheres, but they do not form large clusters or colonies as is the case with *Lactobacillus salivarius*. For this strain, the “number” of bacteria was higher on all surfaces studied in comparison with *Streptococcus sanguinis*. Moreover, *Lactobacillus salivarius* showed the same configuration on the different surfaces, and small agglomerations and short chains were observed (Figure 7b). The size of the Lactobacillus rods was smaller than for *Streptococcus sanguinis* with values of 0.4 to 1.2 micrometers in length of the major axis. The morphologies of both bacterial species were not modified by topography.

**Figure 8:**
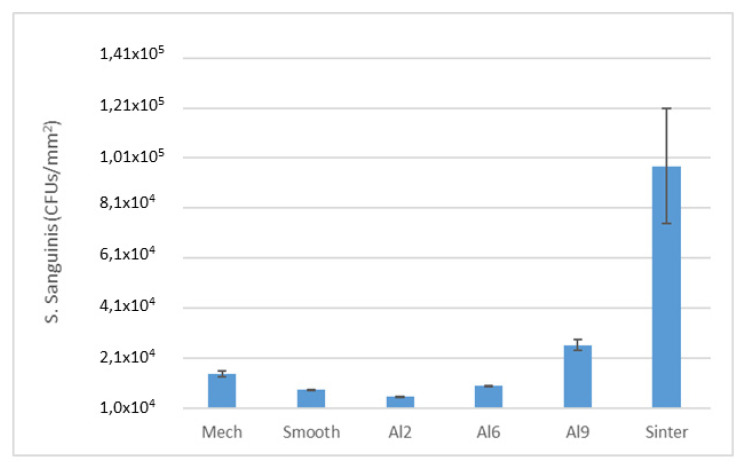
CFUs/mm^2^ of Streptococcus sanguinis for the different treatments studied. Each symbol means that the results were different with statistical significance to the other symbols at *p* < 0.01. For this bacterial strain, the Sinter and Al9 surfaces showed statistically significant differences between them and the rest of the samples. The Al6 and Mech surfaces did not show any differences and neither did the Al2 and Smooth surfaces, but there were differences between the other surfaces.

**Figure 9:**
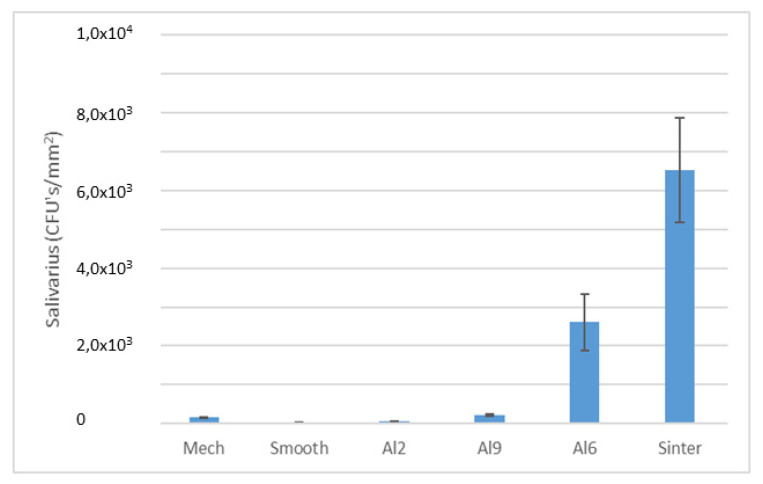
CFUs/mm^2^ of Lactobacillus salivarius for the different treatments studied. Each symbol means that the results were different with statistical significance to the other symbols at *p* < 0.01. For this bacterial strain, the Sinter and Al9 surfaces showed statistically significant differences between them and the rest of the samples. The Al6 and Mech surfaces did not show any differences and neither did the Al2 and Smooth surfaces, but there were differences between the other surfaces.

An evaluation of colony forming units (CFUs) per square millimeter (*p* < 0.005) can be observed in Figure 8 and Figure 9. The two bacteria strains showed a lower tendency to adhere on the Smooth (*Lactobacillus salivarius* ~4.27 × 10^1^/mm^2^*, Streptococcus sanguinis* ~8.02 × 10^3^/mm^2^) rather than rougher surfaces. However, on the rougher surfaces (e.g., Al2), fewer bacteria attached on the two bacteria strains studied (*Lactobacillus salivarius* ~9.73 × 10^1^/mm^2^*, Streptococcus sanguinis* ~5.03 × 10^3^/mm^2^) can be observed at the same time. These results are very significant, as a slightly rough surface, such as Al2, performed slightly better than the polished surface for at least one of the strains studied. That is, sometimes the nanotextures of the surfaces can generate bactericidal behaviors, as they were exposed to different articles when the titanium samples were treated with chemical agents such as Piranha [35]. Rougher samples increased the number of CFUs: Al6 presented few CFUs with *L. Salivarius* (~3.14 × 10^2^/mm^2^), whereas *Streptococcus sanguinis* (~1.03 × 10^4^/mm^2^) showed almost the same number of bacteria attached on Al9 *(Streptococcus sanguinis* ~2.50 × 10^4^/mm^2^), although the quantification with *Lactobacillus salivarius* was higher for Al9 (~2.90 × 10^3^/mm^2^). The sintered samples showed the worst behavior towards both bacterial strains of the surfaces studied. The values obtained for *Lactobacillus salivarius* and *Streptococcus sanguinis* were 6.12 × 10^3^/mm^2^ and 9.59 × 10^4^/mm^2^.

One aspect to be taken into consideration is that the alumina sandblasting treatments were found to have a bactericidal character. This was due to the fact that the alumina residues remaining on the surface caused a change in the surface energy of the titanium as well as its wettability characteristics in the polar and dispersive components, making the surface less favorable to bacterial colonization [38,39,40].

The CFUs cultured on both types of bacteria were determined and compared under the same conditions (because the actual areas on the different discs were not the same). Initially, the results of the CFUs/mm^2^ on the MRS and Todd–Hewitt suspensions showed a correlation with topography (rougher surfaces showed more CFUs/mm^2^, resulting in minimal CFUs/mm^2^ on the Smooth surface) due to the possible effect of the interaction of the bacteria with the rough surface and the hydrophilic and/or hydrophobic character. This hydrophobic tendency was evident in the quantifications of *Streptococcus sanguinis*, where the Smooth and Mech samples showed a high number of CFUs/mm^2^ compared to rougher surfaces such as Al2.

In cellular behavior, prevention of bacterial proliferation plays a key role in implant osseointegration. It has been shown through studies that microbiological infection can produce fibrosis of connective tissue around the implant, mainly via inflammatory reactions, triggering loss of the dental implant [41,42]. Surface properties, such as roughness or surface free energy, are important in bacterial adhesion, formation of biofilms and development of pathologies. Generally, it is explained that bacterial adhesion is favored on roughened surfaces, such as surface valleys, depressions, pits and edges [43], but few studies have analyzed bacterial adhesion on micro- and nano-roughness combined surfaces. These values are in accordance with Amoroso et al., who suggested a lower surface roughness cutoff value (between 34 and 155 nm) for reduced bacterial adhesion [44]. In that work, they confirmed that an increase in the roughness did not improve the attachment of *P. gingivalis*, because the increased size of the surface irregularities was then too large to offer increased bacterial retention. Although smooth surfaces diminished bacterial adhesion, the generation of a biological seal, stimulated by microroughness surfaces, might be more critical for bacterial colonization prevention and the successful integration of the mesh, balancing the race for the surface and greater tissue integration [45,46].

This work presents some limitations, since the study of the microbiological behavior was carried out with only two bacterial strains and no biofilm was produced that would have allowed for a better understanding of the influence of the different topographies. Moreover, we took two types of bacteria, widely used in studies, since they are aerobic and anaerobic, but there are some strains with pathogenesis. Throughout the study, we followed international protocols so as to be able to compare with other investigations.

## 4. Conclusions

Six surfaces with different roughness were studied with the aim of obtaining good fibroblast growth in order to achieve a good biological sealing in the dental mesh. In addition, the osteoblastic capacity was intended to be as small as possible to avoid osseointegration of the mesh. We were able to determine that alumina sandblasted samples of sizes between 212 and 300 μm provide the best compromise between fibroblasts and osteoblasts. Microbiological studies determined that the roughness generated by these particles presents a behavior similar to the polished samples with minimal bacterial colonies on their surface. It was shown that increased roughness leads to increased contact angles by studying wettability and, thus, makes the surfaces more hydrophobic. Furthermore, this treatment showed a low bacterial adhesion (*Streptococcus sanguinis* and *Lactobacillus salivarius*) comparable to polished surfaces. We can also conclude that the increased roughness favored bacterial growth. The meshes obtained by sintering did not show good biological behavior, having the highest cytotoxicity indexes, and their surface favored bacterial colonization. Therefore, this treatment is highly recommended for dental meshes, as it produces a good biological seal, does not favor osseointegration and has excellent behavior against bacteria.

## Figures and Tables

**Table 1:** Chemical composition of the different surfaces analyzed by the dispersive energy of X-rays.

Samples	Al	V	Fe	Si	C	Ti
Mech	0.11 ± 0.12	-	0.23 ± 0.02	-	0.40 ± 0.03	balance
Smooth	0.12 ± 0.23	-	0.21 ± 0.03	0.20 ± 0.02	0.61 ± 0.04	balance
Al2	1.21 ± 0.22	-	0.30 ± 0.04	-	0.71 ± 0.02	balance
Al6	1.41 ± 0.24	-	0.21 ± 0.07	-	0.81 ± 0.07	balance
Al9	1.63 ± 0.35	-	0.32 ± 0.05	-	0.51 ± 0.03	balance
Sinter	6.40 ± 0.52	3.80 ± 0.12	0.51 ± 0.04	-	0.72 ± 0.09	balance

## Data Availability

The authors can provide details of the research requesting by letter and commenting on their needs.
